# Radon Progeny Adsorption on Facial Masks

**DOI:** 10.3390/ijerph191811337

**Published:** 2022-09-09

**Authors:** Annika Hinrichs, Claudia Fournier, Gerhard Kraft, Andreas Maier

**Affiliations:** 1 GSI Helmholtzzentrum Für Schwerionenforschung GmbH, Planckstr. 1, 64291 Darmstadt, Germany; 2 Department of Physics, Goethe Universität Frankfurt am Main, 60438 Frankfurt, Germany

**Keywords:** radon progeny, FFP2 masks, filtration

## Abstract

The radioactive noble gas radon and its short-living progeny are inhaled during respiration, depositing their decay energies in the lungs. These progeny are considered responsible for more than 95% of the total effective dose and are, together with radon, classified as carcinogenic for lung cancer. Consequently, filtration of the progeny could reduce the dose to the lungs. In our study, we investigated the filtration properties of FFP2 versus surgical masks (II R) for radon and its decay products. The masks were attached to a measurement device, which enabled determination of the size distribution of radon progeny, ranging from unattached to clustered progeny. In parallel, it measured the radon activity concentration during experiments. By comparing background measurements without mask and experiments with masks, the percentage of retained unattached radon progeny was determined for FFP2 (98.8 ± 0.6%) and II R masks (98.4 ± 0.7%). For clustered progeny, the retained fraction was 85.2 ± 18.1% for FFP2 and 79.5 ± 22.1% for II R masks while radon was not filtered. We can show that masks are effective in filtering radon progeny and thus are capable of reducing the total effective dose to the lungs.

## 1. Introduction

**Figure 1:**
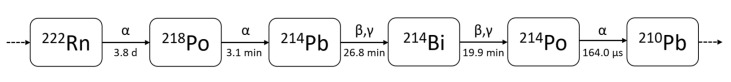
Part of the ^238^U decay chain with the relevant nuclides from ^222^Rn to ^210^Pb with their associated decay modes and half-lives [2].

The naturally occurring radon isotope ^222^Rn that is produced in the decay cascade of ^238^U is a radioactive noble gas. It is the most abundant radon isotope and is responsible for the largest proportion of annual radiation exposure from natural sources [1]. A part of the decay chain is shown in Figure 1. Concerning the human organism, the nuclides relevant for radiation exposure end with ^210^Pb with a half-life of 22.3 years, as removal from the body can occur within this timeframe.

According to this decay scheme, radon-containing atmosphere is a mixture of the primary radon and the decay products that can attach to aerosol particles [3]. Inhalation is the primary route of incorporation, where the solid radon progeny can deposit in the airways. This can happen either by inhalation of airborne progeny or by inhaled radon, which will decay while passing through the respiratory tract. In this process, the α-emitting polonium isotopes ^218^Po and ^214^Po are the largest contributors to the dose, with a decay energy of 6.00 MeV and 7,69 MeV, respectively, while the β- and γ-emitting isotopes contribute to about 10% [4,5]. In contrast, most of the ^222^Rn is exhaled immediately [6]. As the progeny will largely deposit on the surface of the respiratory tract, the lung equivalent dose is considered to contribute to more than 95% of the total effective dose [7]. Therefore, radon and its decay products are classified as carcinogenic for lung cancer in humans [8].

Shortly after their α-decay, most of the remaining nuclei are positively charged as orbital electrons are evaporated [9]. In a first step, these nuclei will react with trace gases and water vapor in a so-called clustering process, which will take less than a second. Due to their small size (0.5–5 nm), these particles will have a high diffusion coefficient [3] and are called unattached progeny [1]. In the next 1–100 s, these clusters will attach to aerosols, forming clustered (20–100 nm) and attached (>100 nm) progeny [3]. The adhesion probability between the progeny and an aerosol can be assumed to be 100% [10].

Besides these processes, the adhesion of these different size fractions to surfaces plays an important role. The following three deposition mechanisms are of relevance and can be distinguished, depending on characteristics such as size and shape and the physiological parameters of the airway system. For particles with a diameter of 2–20 μm, inertial impactions play the major role, as they keep their trajectory despite changes in direction of air stream because of their inertial momentum. The respective particles deposit mainly in the upper region of the respiratory tract.

The second process is sedimentation of particles between 0.1–50 μm, which settle due to gravity. This process takes place in the upper respiratory tract and mainly in bronchioles and alveoli. The last process is diffusion due to Brownian motion for particle sizes <0.2 μm, dominating in the gas-exchange region and in the upper respiratory tract because the diffusion coefficient increases with decreasing particle size. As particles in the medium size range from 0.2–0.5 μm are too lightweight for sedimentation but have a decreased diffusion coefficient, the total lung deposition shows a minimum of these particles [11,12,13,14].

For the determination of primary radon, different measurement techniques are used, such as electronic devices, CR-39 or an electret method [15]. Additionally, radon can be adsorbed in charcoal [16], where it can bind via Van der Waals interaction and can be determined with spectroscopic methods due to its decay [17].

Other studies have revealed that filtration can significantly reduce the dose up to almost 70% in the respiratory system, depending on the filtering method. Typical methods are HEPA filters, ion generator/fan systems and electrostatic air cleaners [18,19,20]. This bears the possibility of a decrease in the lung cancer risk that is related to radon exposure, as radon progeny contribute to a major part of the total effective dose in the lungs.

As described, reducing the effective dose to the lungs is achieved by air filtering accompanied by installing special devices. However, individual protection steps to reduce the lung dose may be a possibility, especially if these devices are not yet present. Due to the COVID-19 pandemic, the wearing of face masks (FFP2 or surgical masks) as a safety measure is recommended by the WHO [21].

In this study, we aim to investigate whether this simple method of wearing a face mask is capable of reducing the lung dose. Therefore, we experimentally determined the filtration properties of these masks for radon progeny. As different mask types reveal a different structure and different filtering properties, both types were tested and compared. Wearing these masks may be an easy way to reduce lung dose, especially in basements with low ventilation and consequently high radon activity concentrations, as well as for staff working in radon therapy.

## 2. Materials and Methods

The experiments were performed in an in-house-designed radon chamber, where reproducible exposure conditions could be administered. Besides temperature and relative humidity, the radon activity concentration could be adjusted and permanently monitored [22]. For the measurements of radon progeny adsorption, two types of filters were used:

- Melt-blown protective mask (IPOS—Medikal, Diş Ticaret A. Ş, Istanbul, Turkey), consisting of nonwoven fabric on the outer and inner layers and spunbond fabric on the middle layer, with a performance level of FFP2 and in conformity with the EU-type examination certificate 224-21-01-R02; thickness: 0.9 ± 0.1 mm (referred to as FFP2); test standard: EN 149:2001 + A1:2009.
- Surgical mouth and nose protection mask type II R (IPOS—Medikal, Diş Ticaret A. Ş, Istanbul, Turkey), consisting of nonwoven fabric; thickness: 0.5 ± 0.1 mm (referred to as II R); test standard: DIN EN 14683:2019-10.

**Figure 2:**
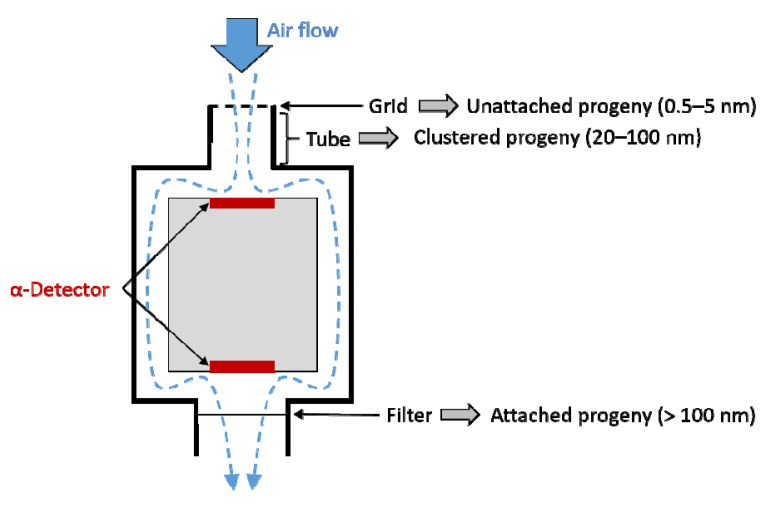
Schematic drawing of the sampling head used to measure radon and radon progeny (EQF 3220, Sarad GmbH, Dresden, Germany) with size fractions assigned to their component where they attach. Air passes through the device, and unattached progeny are retained in the grid, while clustered progeny mainly remain stuck at the tube and attached progeny at the filter, respectively.

For the measurements of the radon progeny adsorption, a commercially available measurement device (EQF 3220, Sarad GmbH, Dresden, Germany) was used. This device enables the detection of radon activity concentration during exposure. In parallel, it can distinguish radon progeny in relation to their size distribution, ranging from unattached (<5 nm) and clustered (20–100 nm) to attached (>100 nm) progeny, with a sampling head for measuring the α-emitting isotopes ^218^Po and ^214^Po via α-spectroscopy. Air passes through the sampling head with a defined flow rate. According to size, particles attach to different components. Due to their high diffusion coefficient, particles of the unattached progeny attach to the grid at the beginning of the device. Bigger particles of the clustered progeny mainly stick to the inside of the tube. Discriminating between the two fractions is based on differences in the α-particle energy due to their different air path, meaning the distance from the place of decay to the detector. However, the clustered progeny partly attach to the back face of the grid and the detector. These differences in attachment of the same size fraction are considered and included in the calculations, which leads to a higher error of measured values for the clustered fraction. The attached fraction is filtered at the end of the sampling head and detected with a separate α-detector (Figure 2).

The detector head was placed in the middle of the radon chamber with a distance of 22.50 cm to the wall, 1.85 cm to the bottom and 31.85 cm to the top, respectively. Throughout the experiments, a constant flow of 1.49 L min^−1^ through the detector head was maintained. For the experiments, the detector head was placed in the chamber, and pure radon was introduced. The measurement was started in parallel to the introduction of ^222^Rn into the chamber, with a measurement interval of three minutes. The typical total measurement time was up to 5 h, allowing radon to receive radioactive equilibrium with its daughter nuclei ^218^Po, ^214^Pb, ^214^Bi and ^214^Po after approximately 3 h. While the amount of radon content is given as activity concentration in the unit Bq m^−3^, the radon progeny is given as equilibrium equivalent concentration (EEC) for each size fraction separately. The EEC is characterized as the virtual radon activity concentration of an assumed atmosphere in equilibrium that would have the same potential alpha energy concentration (PAEC) as the existing nonequilibrium mixture. The PAEC is defined as the concentration of short-lived radon progeny in the air in terms of the alpha energy emitted during complete decay from ^218^Po to ^210^Pb in a unit volume of air [23]. The EEC is also given in the unit Bq m^−3^. Values for EEC and radon activity concentration were obtained simultaneously.

First, experiments were performed with varying radon activity concentrations between 88–375 kBq m^−3^. After 180 min, the clustered and unattached fractions were determined in order to prove whether there was a linear relationship between the radon activity concentration and the EEC of these fractions at the same time (linearity experiment).

**Figure 3:**
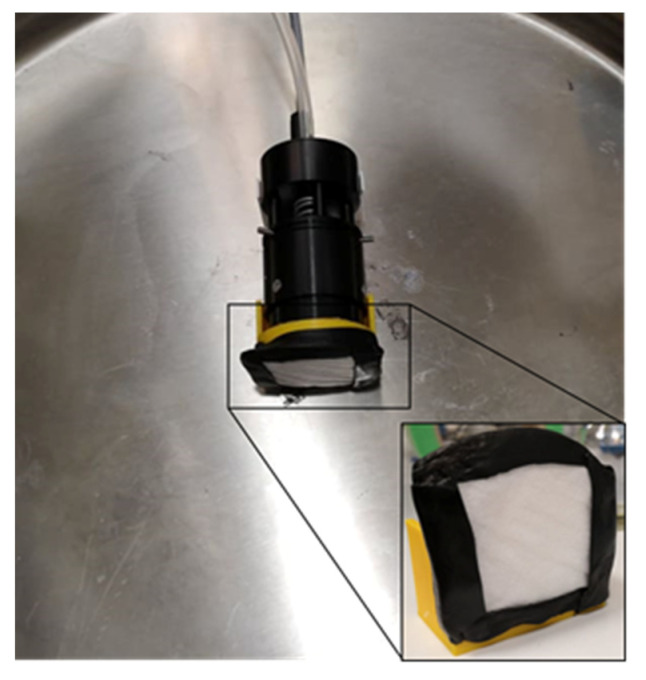
Measurement setup with detector head, to which the filter was attached via cellular rubber. The entire device was placed inside the radon chamber.

Further experiments for radon progeny adsorption were performed with FFP2 and II R masks. They were cut to square with a side length of 4.5 cm and hermetically attached with cellular rubber to a 3D-printed mounting and the detector head (see Figure 3). All experimental conditions can be found in Table 1. Unless stated otherwise, all given values are mean values with their corresponding standard deviation.

For analysis, only data 180 min after radon induction were taken into account, as the radioactive equilibrium was reached after this time. For each measurement interval, the EEC per size fraction was normalized according to the radon activity concentration. From these values, the mean value was determined for each independent experiment without a filter and with FFP2 and II R masks.

By comparing the data of the FFP2 and II R masks with the measurements without a filter, the percentage of retained radon progeny was obtained for the unattached and the clustered fractions. The attached fraction was not considered any further, as this fraction was negligibly small in our experimental setup.

To show that primary radon will not be adsorbed on any type of mask, the radon activity concentration during experiments was additionally determined with a second radon measurement device (RTM 1688-2, Sarad GmbH, Dresden, Germany). It measured the radon activity concentration in the radon chamber at intervals of three minutes by aspiration of the air into the instrument. In all experiments, there was no mask attached to the air inlet. The measured radon activity concentration from the two separate measurement devices can be compared at the same time points. One device was measuring without a filter in every experiment (RTM 1688-2), and the other one without (no filter) or with a filter (FFP2 or II R, EQF 3220), revealing no difference between the experiments. This excluded radon adsorption by the different masks.

## 3. Results

**Figure 4:**
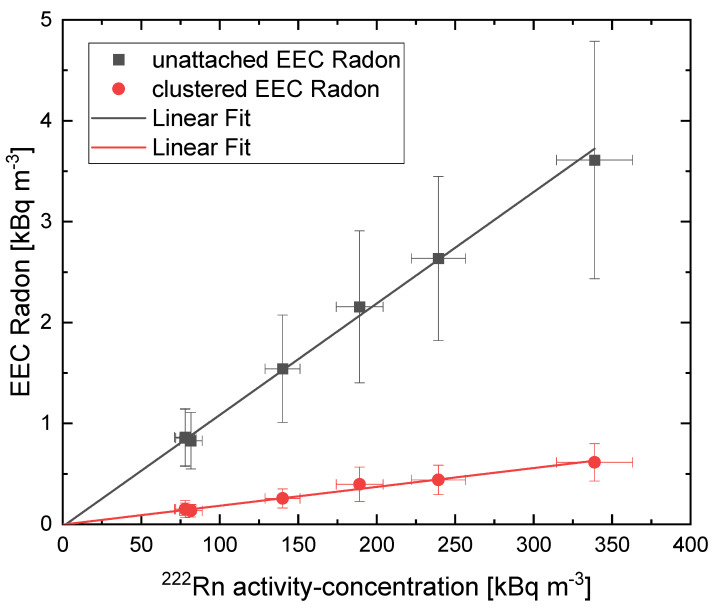
Relationship between radon activity concentration and unattached (grey) and clustered (red) equilibrium equivalent concentrations. The solid line represents a linear fit; the uncertainty was weighted by 1 σ^−2^, with σ being the size of the error bar obtained by the measurement device. The fit was performed using the software Origin. It represents a linear regression with an R^2^ of 0.99 for the unattached fraction and R^2^ of 0.98 for the clustered fraction, confirming a linear relationship.

First, the experiments confirmed a linear relationship between the EEC of both size fractions and the radon activity concentration. This not only allowed normalization to this parameter, but additionally, the results are independent of applied radon activity concentration. The results are shown in Figure 4.

**Figure 5:**
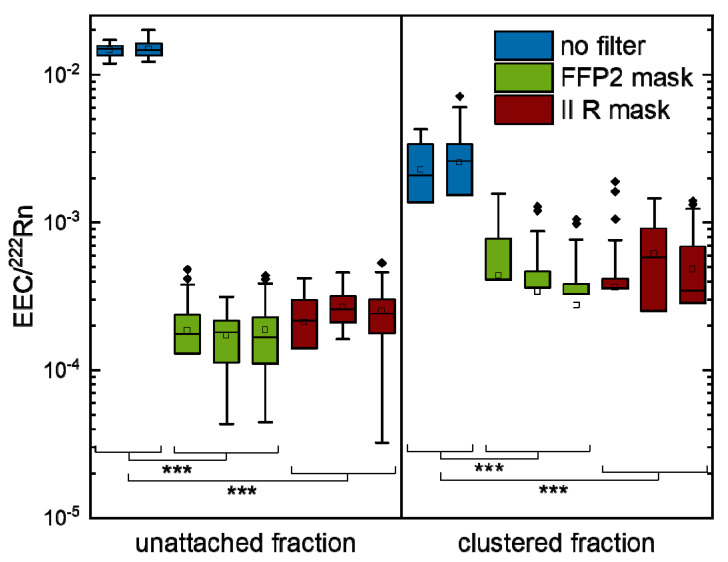
EEC of unattached and clustered fractions in relation to the radon activity concentration at the same time for measurements without filter and for the individual experiments with FFP2 mask. The box describes the values from the first to the third quartile, the whisker the 1.5 interquartile range, the horizontal line depicts the median, the cube the mean and diamonds outliers (no filter: N = 2, *n* = 65; FFP2: N = 3, *n* = 122; II R: N = 3; *n* = 122). Significance was tested with Welch’s test. *p*-values are indicated by asterisks, with * for *p* ≤ 0.05, ** for *p* ≤ 0.01 and *** for *p* ≤ 0.001.

To determine the amount of radon progeny in the different size fractions, the values for the EEC in the unattached and clustered fraction were normalized to the respective radon activity concentration for all time points ≥ 180 min after radon induction. Afterwards, the values were summarized for each experiment. The results for each of the three experiments with FFP2 and II R masks and the two experiments without a filter are depicted in Figure 5, showing a good internal reproducibility. In addition, the numerical values for each experiment are summarized in Table 2. As we could not detect any attached fraction, either with or without filter, this part was excluded from further analysis and is not shown here.

**Figure 6:**
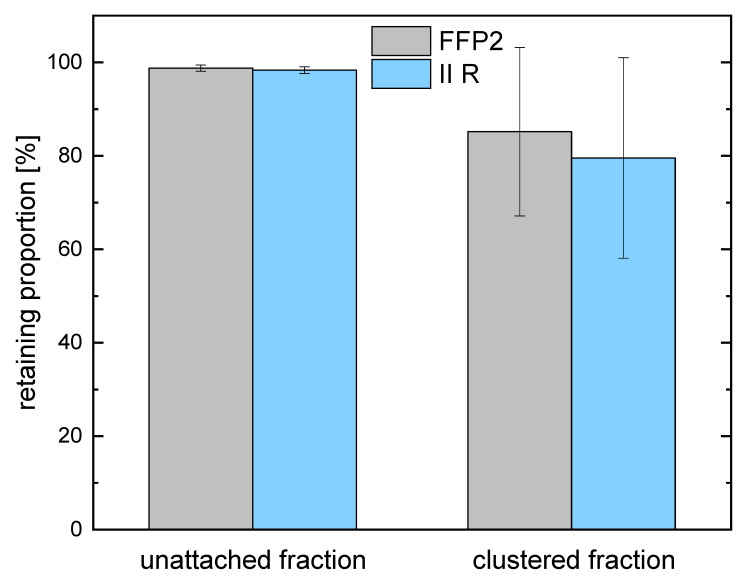
Retaining proportion of unattached and clustered fractions for FFP2 and II R masks in comparison to the measurement without a filter. The upper edge corresponds to the mean value, and errors are calculated by Gaussian error propagation.

The values for the individual measurements were combined for each mask type and size fraction, and the percentage of retained radon was obtained by comparing them with the results from the measurements without a filter. The results are shown in Figure 6 and Table 3.

## 4. Discussion

With our measurements, we could show the filtration properties of II R and FFP2 masks for radon progeny in the unattached and clustered fractions. Filtering of radon progeny with commercially available filters can reduce the activity concentration in the range of 60–80%. For the purpose or filtration, the unattached fraction is an especially critical factor in the risk evaluation, as this size fraction can deposit very effectively in the respiratory tract [19].

Therefore, face masks seem to be very effective in reducing the activity concentrations. Particles with sizes <200 nm are deposited by Brownian motion [11,12] and can be described by gas kinetic laws [24], whereas electrostatic forces can be neglected [3].

The diffusion rate for different particle sizes can be calculated by:

(1) $$v=\sqrt{\frac{3k_{B}T}{m}}$$

where *v* is diffusion rate, *k_B_* is Boltzmann‘s constant (1.380649·10–23 J K^−1^), *T* is absolute temperature, and *m* is the mass of the particle. For the mass, spheres with diameters of 0.5, 5, 20 and 100 nm and a density of 1 g cm^−3^ are assumed. The results are summarized in Table 4.

As the flow rate through the sample is determined by the volume flow of 1.49 L min^−1^ and the area of 1.33 cm^2^ of the detector head, a flow rate of 0.19 m s^−1^ can be obtained. As a result, the diffusion rate for a particle with a diameter of 100 nm is almost the same as the flow rate through the sample, whereas it is around 2300 times larger for a diameter of 0.5 nm. Therefore, it is much more likely that particles with a smaller size will adsorb on the surface of the fibers of the mask than for particles with bigger diameters.

However, our results for the unattached and clustered fractions were averaged over the entire range of 0.5–5 nm or 20–100 nm, respectively. Within these fractions, there are large differences in the diffusion rates within the boundaries. With our setup, we could not discriminate the size distribution within the different fractions. We could only assume that smaller particles would adsorb more effectively than larger particles, as long as they were in the range where Brownian motion is the main deposition mechanism.

For our measurements, the different mask samples were perfectly sealed to the measurement setup. Unfortunately, this is not the case for real operation, as especially II R masks do not ensure a good hermetical seal, and they allow particles to enter around the edges [25].

For FFP2 masks, the initial fit pass rates are between 60–95%, depending on different factors, such as age, weight, face length and width, lip and nose length. Moreover, facial hair will influence the sealing of these masks [26]. Therefore, higher leakage rates will lower the filter efficiency for radon progeny. A quantitative analysis for this factor is out of scope for this paper.

Additionally, it is recommended that FFP2 masks can be worn for up to 4 h [27]. After this time, the filtration efficiency will also decrease due to moisture. In our experiments, the setup was exposed for 5 h at a relative humidity of around 65%, which corresponds to the mean natural relative humidity in the environment, giving no hint of a reduced efficiency during the end of the measurement. However, exhaled air has a relative humidity of around 100%, which may influence the filter material. Therefore, our results presented here are peak values, which may be reduced for real-life application.

In our experiments, no conclusion on attached progeny with a size >100 nm could be drawn. However, for FFP2 masks, a filtration capacity of 95% for particle sizes of 300 nm are mandatory [25]. This gives rise to the assumption that FPP2 masks will also effectively filter the attached progeny.

## 5. Conclusions

Our results provide solid guidance that face masks will significantly reduce the activity concentration of radon progeny in the airway system, whereas radon is not filtered. Nevertheless, filtering can lead to lower doses to the lungs during radon exposure and thus to a reduced risk for lung cancer.

Besides exposure of the general public, this is also important for occupational exposure, for example in radon galleries or radon baths, where patients suffering from inflammatory diseases such as rheumatoid arthritis are treated [14]. In these treatment facilities, enhanced levels of radon and progeny can be measured, making efficient ventilation necessary [28,29]. Therefore, the wearing of facial masks may be an easy and cost-efficient method for dose reduction in the staff.

## Figures and Tables

**Table 1:** Experimental conditions during measurements for linearity experiments, without filter (no filter), and with FFP2 mask and II R mask. The range of the radon activity concentration is described as c(Rn).

Experiment	Temperature	Relative Humidity	c(Rn)
linearity	21.9 ± 0.3 °C	74.6 ± 2.8%	88–375 kBq m^−3^
no filter	22.4 ± 0.1 °C	63.9 ± 2.3%	19.7–54.5 kBq m^−3^
FFP2	22.2 ± 0.1 °C	65.8 ± 1.7%	54.5–79.1 kBq m^−3^
II R	22.1 ± 0.2 °C	67.4 ± 2.5%	75.8–104.4 kBq m^−3^

**Table 2:** Summary of individual experiments without a filter and with FFP2 and II R masks. Values represent the EEC normalized to the radon activity concentration for the unattached and the clustered fractions.

Experiment	Unattached Fraction	Clustered Fraction
no filter 1	(1.47 ± 0.13)·10^−2^	(2.28 ± 1.15)·10^−3^
no filter 2	(1.50 ± 0.20)·10^−2^	(2.54 ± 1.92)·10^−3^
FFP 2_1	(1.86 ± 1.01)·10^−4^	(4.37 ± 4.28)·10^−4^
FFP 2_2	(1.72 ± 0.75)·10^−4^	(3.42 ± 3.65)·10^−4^
FFP 2_3	(1.87 ± 0.99)·10^−4^	(2.77 ± 2.98)·10^−4^
II R 1	(2.11 ± 1.01)·10^−4^	(3.66 ± 4.20)·10^−4^
II R 2	(2.68 ± 0.74)·10^−4^	(6.11 ± 4.13)·10^−4^
II R 3	(2.50 ± 1.11)·10^−4^	(4.83 ± 3.81)·10^−4^

**Table 3:** Retained proportion for each size fraction and different mask.

Experiment	Unattached Fraction	Clustered Fraction
FFP2	98.77 ± 0.64%	85.17 ± 18.05%
II R	98.36 ± 0.69%	79.54 ± 21.47%

**Table 4:** Diffusion rate for spheres with different diameters and a density of 1 g cm^−3^.

Particle Diameter	Diffusion Rate
0.5 nm	432.18 m s^−1^
5 nm	13.67 m s^−1^
20 nm	1.71 m s^−1^
100 nm	0.15 m s^−1^

## Data Availability

The data presented in this study are available on request from the corresponding authors.
